# Efficacy and safety of Tai Chi for chronic musculoskeletal pain: a systematic review and meta-analysis

**DOI:** 10.3389/fpain.2026.1678660

**Published:** 2026-02-24

**Authors:** Shun Chen, Weiting Liu, Qinwei Fu, Mingyu Huang, Weilan Lin, Yanting Ding, Ming Li

**Affiliations:** 1 The First School of Clinical Medicine, Fujian University of Traditional Chinese Medicine, Fuzhou, China; 2 School of Nursing and Midwifery, Edith Cowan University, Perth, WA, Australia; 3 JBI Affiliated Centre for Evidence Informed Nursing, Midwifery and Health Care Practice, Edith Cowan University, Perth, WA, Australia; 4 Research Centre of Prevention and Management of Chronic Disease, Edith Cowan University, Perth, WA, Australia; 5 School of Clinical Medicine, Chengdu University of Traditional Chinese Medicine, Chengdu, China; 6 Department of Massage of Main Hospital, Xiamen Hospital of Traditional Chinese Medicine, Xiamen, China; 7 Department of Massage, Tai'an Hospital of Traditional Chinese Medicine, Tai'an, China; 8 College of Integrated Traditional Chinese and Western Medicine, Ji’ning Medical University, Ji’ning, China

**Keywords:** chronic musculoskeletal pain, efficacy, safety, systematic review, Tai Chi

## Abstract

**Objectives:**

Tai Chi, a form of complementary intervention emphasizing breathing and mind-body connection, is increasingly recognized for its potential in pain management. This review aims to synthesize current evidence on the efficacy and safety of Tai Chi for chronic musculoskeletal pain management in adults.

**Design:**

Systematic review and meta-analysis.

**Data sources:**

AMED, EMBASE, MEDLINE, WoS, CBM, CNKI, VIP, Wanfang Databases, CENTRAL, and WHO ICTRP were searched from database inception through May 2025, with an additional supplementary search conducted in January 2026.

**Eligibility criteria for selecting studies:**

We included randomized controlled trials (RCTs) comparing Tai Chi with other interventions for chronic musculoskeletal pain management. The primary outcome was the efficacy of Tai Chi, and secondary outcomes were adverse events associated with Tai Chi for chronic musculoskeletal pain.

**Data extraction and synthesis:**

Two independent authors screened studies, extracted data, and assessed the risk of bias using the Cochrane Risk of Bias Tool 2. Meta-analysis was performed using RevMan 5.3 software, presenting results with 95% confidence intervals (CI) and Standard Mean Difference (SMD). The certainty of evidence for primary outcomes was evaluated using the Grading of Recommendations, Assessment, Development, and Evaluation (GRADE) criterion approach.

**Results:**

Thirty-eight RCTs were included in this review. Compared to control groups, Tai Chi was associated with reduced pain in osteoarthritis [SMD = −0.37, 95% CI (−0.57, −0.16)], low back pain [SMD = −1.43, 95% CI (−2.07, −0.80)], fibromyalgia [SMD = −0.96, 95% CI (−1.96, 0.04)], and other disease [SMD = −1.04, 95% CI (−2.14, 0.06)]. Most interventions lasted 12 weeks, and Yang-style Tai Chi and 24-form Tai Chi were most frequently studied. No serious Tai Chi–related adverse events were reported.

**Conclusion:**

Tai Chi appears to be associated with pain reduction in osteoarthritis and low back pain, while effects in fibromyalgia and other musculoskeletal conditions were non-significant, and no serious adverse events were reported in the included trials. The certainty of evidence is limited by methodological limitations and risk of bias; therefore, the findings should be interpreted with caution.

**Systematic Review Registration:**

https://www.crd.york.ac.uk/PROSPERO/view/CRD42023426431, Identifier CRD42023426431.

## 1 Introduction

Chronic musculoskeletal pain is one of the most prevalent and disabling forms of chronic pain (1), often resulting from or associated with conditions such as osteoarthritis, low back pain, and fibromyalgia. Global burden studies confirm that musculoskeletal disorders are among the leading causes of disability worldwide, and a growing global burden of chronic musculoskeletal pain in middle-aged adults, with projections pointing to a continued rise through 2050 (2, 3). Chronic musculoskeletal pain not only affects mobility and functional capacity but also contributes significantly to reduced quality of life, productivity loss, and increased healthcare utilization (4, 5). A recent National Institutes of Health report has shown that nearly two-thirds (61.4%) of individuals with chronic pain continue to experience it a year later (6), underscoring its persistence and social burden, and the critical need for effective pain management strategies.

Non-steroidal anti-inflammatory drugs are widely regarded as first-line pharmacological options for pain management. However, the use of such drugs is not without risks, including physiologic dependence, addiction potential, and heightened susceptibility to conditions like coronary heart disease and gastric (7). Thus, the quest for effective non-pharmacological approaches to chronic musculoskeletal pain management is of paramount importance and warrants urgent attention. Recent international guidelines, including the World Health Organization (2023) guideline for the management of chronic low back pain (LBP) and the National Institute for Health and Care Excellence (2021) for the chronic pain recommendations, emphasise non-pharmacological strategies such as exercise, mind–body practices, and psychological therapies as preferred options for chronic pain management, reflecting a global shift toward evidence-based and holistic care (8, 9).

Tai Chi, also known as Tai Ji Quan, is a gentle to moderate physical exercise with a rich historical practice, recognized both in Eastern and Western cultures (10). Tai Chi is characterized by slow, smooth movements with graceful transitions, synchronized with deep and regular breathing, and a focus on relaxation (11). Increasing evidence suggests that Tai Chi may improve musculoskeletal pain by enhancing muscle strength, flexibility, and proprioception, while also reducing stress and anxiety (12, 13). In recent years, Tai Chi has garnered significant research attention, particularly in chronic pain management, with several systematic reviews published (14–17). However, previous reviews provided limited evidence. Peng et al. (14) included 10 randomized controlled trials (RCTs) but did not perform subgroup or meta-analysis, which restricted quantitative synthesis. Kong et al. (15) reviewed 18 RCTs, yet subgroup analyses were based on only two to three studies, resulting in limited statistical power. Another study (16) focused on chronic musculoskeletal pain but included a relatively small number of studies and did not assess safety outcomes. Zou et al.'s study (17) combined multiple mindful exercises, making it difficult to isolate the specific effects of Tai Chi. All four previous systematic reviews were constrained by methodological and analytical limitations, including small sample sizes, heterogeneous Tai Chi interventions without differentiation among styles or practice parameters, insufficient subgroup or sensitivity analyses, and limited reporting of safety outcomes, which together reduce the robustness and generalisability of their conclusions.

To address the gap, this systematic review aims to provide an updated and comprehensive evaluation of the existing literature to explore the efficacy and safety of Tai Chi in managing chronic musculoskeletal pain in adults. By refining the scope and applying rigorous analytic methods, this study seeks to clarify Tai Chi's therapeutic role in musculoskeletal pain management and inform future clinical practice.

## 2 Methods

The Preferred Reporting Items for Systematic Reviews and Meta-Analysis (PRISMA) framework (18) was utilized to structure this review.

### 2.1 Eligibility criteria

The inclusion criteria were developed based on key elements of participants, interventions, comparators, outcomes, study design, and time periods. Participants: included adults diagnosed with chronic musculoskeletal pain according to the International Association for the Study of Pain diagnostic criteria (1), as pain persisting for more than 3 months and constituting the sole or a leading complaint. No restrictions were placed on gender or race. Interventions: encompassed all types of Tai Chi, including various movements and forms of these interventions. Comparators: included no treatment, placebo, other non-pharmaceutical therapies, or any conventional treatments for chronic musculoskeletal pain. Outcomes: primary outcome was the pain intensity measured by Visual Analogue Scale (VAS) or Numerical Rating Scale (NRS); when unavailable, a single disease-specific pain measure [e.g., Western Ontario and McMaster Universities Osteoarthritis Index (WOMAC) pain or Fibromyalgia Impact Questionnaire (FIQ) pain] was used according to a prespecified hierarchy. Secondary outcomes were safety, measured by adverse events (AEs) and their potential reasons. Intervention characteristics (intervention durations, Tai Chi types and Tai Chi forms) were examined as prespecified exploratory effect modifiers using subgroup analyses. Study design: included RCTs; and Time periods: considered all lengths of treatment and follow-up duration, prioritizing outcomes reported at the longest time point. Exclusion criteria were as follows: (1) studies lacking information on pain duration; (2) trials combining Tai Chi with other treatments; (3) clinical trial protocols; and (4) inaccessible full-text literature.

### 2.2 Information sources

The Allied and Complementary Medicine Database (AMED), Excerpta Medica Database (EMBASE), Medical Literature Analysis and Retrieval System Online (MEDLINE), Web of Science (WoS), Chinese Biological Medicine Database (CBM), China National Knowledge Infrastructure (CNKI), Chinese Technical Periodicals (VIP), and Wanfang Database were searched. Additionally, Cochrane Central Register of Controlled Trials (CENTRAL) and World Health Organization International Clinical Trial Registration Platform (WHO ICTRP) were consulted to identify ongoing and recently completed studies.

### 2.3 Search strategy

The search strategy employed a combination of controlled vocabulary (MeSH or Emtree, where applicable) and free-text keywords using Boolean operators (AND, OR, NOT). The initial search was conducted in May 2025 using the terms related to chronic pain and Tai Chi, including “pain”, “chronic pain”, “Tai Chi”, “Tai Ji”, and “mind-body therapy”, covering all records from database inception to the search date. Following study selection, the included evidence was found to predominantly address chronic musculoskeletal pain conditions. In line with this refined focus and in response to reviewer feedback, a supplementary search was conducted in January 2026 using musculoskeletal pain–specific terms to ensure comprehensive coverage. Studies published in English or Chinese were considered for inclusion due to feasibility constraints. The detailed search strategies and results for databases are available in Supplementary Appendix 1.

### 2.4 Selection process

Following the initial search, two authors (SC and Q-W F) independently conducted title and abstract screening, excluding irrelevant records. Subsequently, full-text articles were assessed based on eligibility criteria. Agreement on inclusion was reached among all authors after thorough evaluation, with reasons for exclusion documented. Any discrepancies were resolved through consultation with a third author (W-T L). All records were managed and organized using EndNote 21 for screening and reference management.

### 2.5 Data collection process

Data extraction utilized Excel spreadsheets (Microsoft Excel version 2019), with two authors (SC and Q-W F) independently extracting data from the included studies. The extracted information was then evaluated and cross-checked. Inconsistencies were discussed and resolved by involving a third author (W-T L) for consensus. The data extraction procedure was designed to capture key study characteristics, intervention details, and outcome measures comprehensively and systematically.

### 2.6 Data items

From each included study, the following data were extracted: (1) study characteristics: first author, year of publication, country, and sample size; (2) participant characteristics: age and types of chronic musculoskeletal pain; (3) intervention details: Tai Chi style, forms, intervention duration, and follow-up period; (4) comparator details: type and description of control interventions; (5) outcomes: primary outcome (pain intensity, with corresponding measurements), and secondary outcomes (adverse events); (6) other information: funding sources and potential conflicts of interest, when available.

### 2.7 Risk of bias assessment

The quality assessment was independently evaluated by two independent authors (Q-W F and M-Y H) using version 2 of the Cochrane Risk of Bias Tool (19). Any disagreements were further discussed towards consensus with a third author (W-T L). We allocated domains as follows: “randomization process”, “deviations from the intended interventions”, “missing outcome data”, “measurement of the outcome”, “selection of the reported result”, and “overall bias” as “high risk”, “some concerns”, and “low risk”.

### 2.8 Effect measures

For continuous outcomes, the standardized mean difference (SMD) with 95% confidence intervals (CI) was calculated as the effect size for variations in pain assessment tools. This approach allowed for the polling of results across studies employing different measurement scales and ensured comparability of effect estimates. The use of SMD followed recommendations outlined in the Cochrane Handbook for Systematic Reviews of Interventions (20). When identical scales were used, mean differences (MD) were applied. When key data were missing, study authors were contacted to obtain additional information.

### 2.9 Synthesis methods

Meta-analysis was performed using RevMan 5.3 software. Statistical heterogeneity was assessed through *I*^2^ statistics and *Q*-tests, with forest plots visually presenting heterogeneity patterns. The *I*^2^ value and *Q*-test *p*-value were employed to determine heterogeneity levels. Given the observed heterogeneity in pain assessment scales across studies, a random-effects model with SMD was uniformly applied. When low heterogeneity was observed (*I*^2^ < 50% or *p* > 0.01), the results were considered reliable. In cases of substantial heterogeneity (*I*^2^ ≥ 50% or *p* < 0.01), findings were deemed unreliable, prompting sequential exclusion of high-heterogeneity studies until the criteria (*I*^2^ < 50% or *p* > 0.01) were met.

To address unit-of-analysis issues, each trial contributed only one independent effect estimate per outcome; multi-arm trials and same-type control groups were combined, multiple pain measures were reduced to a single prespecified outcome (see Section 2.1 eligibility criteria), and different control types were handled by prespecifying one control group for the primary analysis, in accordance with the Cochrane Handbook (20). Prespecified subgroup analyses were conducted to examine whether the primary effect of Tai Chi differed across disease categories and control types (active vs. inactive), with active controls defined as other exercise or therapeutic interventions and inactive controls defined as waiting list, routine care, or no-treatment comparators. The intervention characteristics (duration, Tai Chi type, and Tai Chi forms) were examined in separate exploratory subgroup analyses.

### 2.10 Reporting bias assessment

Reporting bias was assessed through visual inspection of funnel plot symmetry and statistical tests (Egger's and Begg's tests) when ≥10 studies were included. For analysis with fewer studies, potential reporting bias was evaluated narratively based on study characteristics and publication sources.

### 2.11 Certainty assessment

The certainty of evidence for primary outcomes was assessed using the Grading of Recommendations, Assessment, Development, and Evaluation (GRADE) approach (21), facilitated by the GRADEpro Guideline Development Tool (https://gradepro.org/).

### 2.12 Protocol registration and deviations

The review protocol was registered in the Prospective Register of Systematic Reviews (No. CRD42023426431). During the review process, several protocol refinements were made to improve methodological appropriateness and transparency. Although the original search strategy focused on chronic pain, a supplementary search was conducted to explicitly capture chronic musculoskeletal pain disorders after the scope of the review was refined. Risk of bias was assessed using the Cochrane RoB 2 tool, and data synthesis was performed using RevMan 5.3 rather than ROB 1 and R software. The intervention characteristics (duration, form, and type of Tai Chi) recorded in the protocol were not analysed as main outcomes but were examined in exploratory subgroup analyses.

## 3 Results

### 3.1 Study selection and characteristics

**Figure 1:**
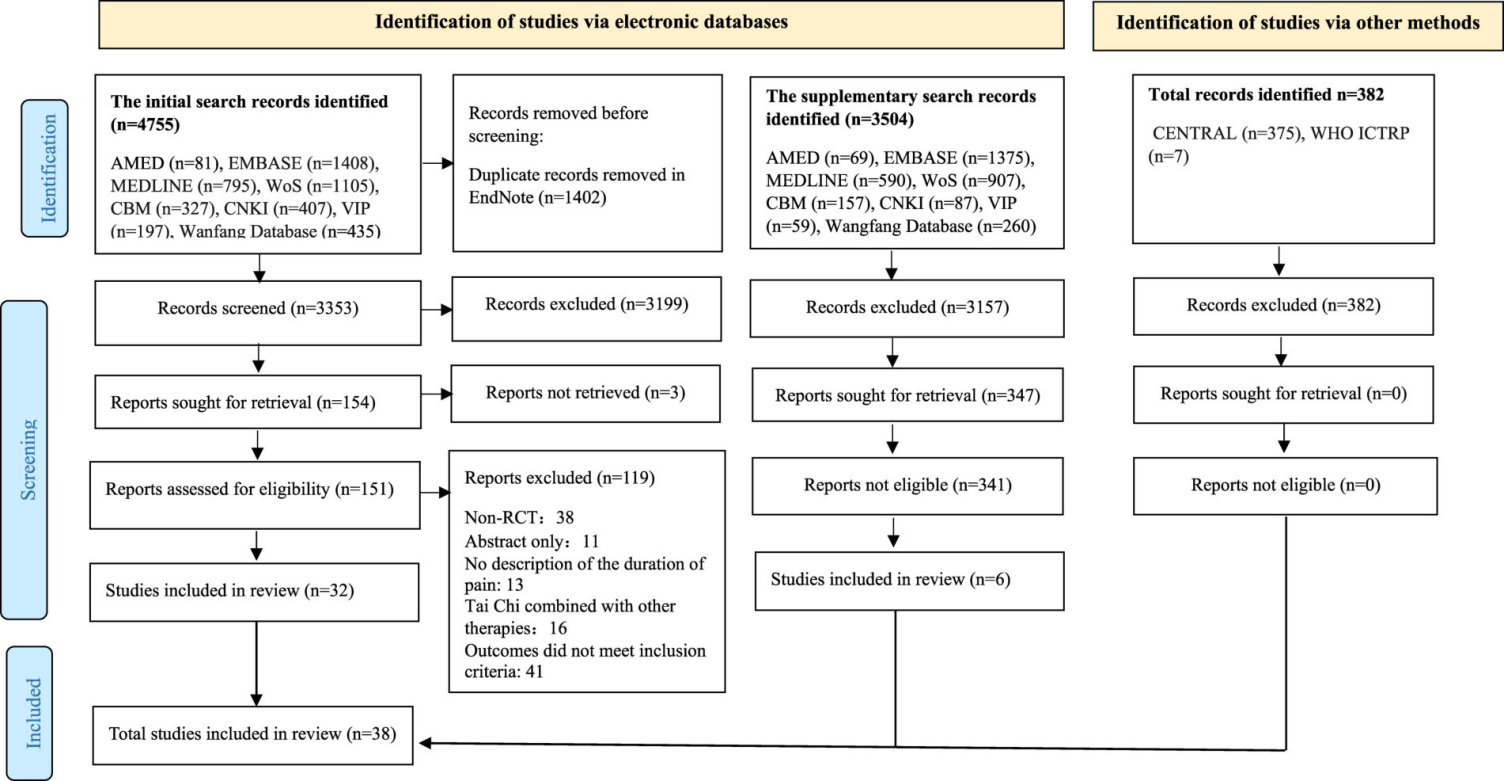
PRISMA diagram. CBM, Chinese biological medicine database; CENTRAL, Cochrane central register of controlled trials; CNKI, China National Knowledge Infrastructure; EMBASE, excerpta medica database; MEDLINE, medical literature analysis and retrieval system online; RCT, randomized controlled trials; VIP, Chinese technical periodicals; Wanfang, Wanfang database; WoS, web of sciences; WHO ICTRP, World Health Organization International Clinical Trials Registry Platform.

A PRISMA flow diagram illustrating the search and screening process is presented in Figure 1. This review encompassed 38 studies, published in both English (*n* = 25) and Chinese (*n* = 13). The study period ranged from 2000 to 2024. Among these studies, 19 were conducted in China (22–40), 12 in the United States (41–52), two in Korea (53, 54), two in Australia (55, 56), one in Turkey (57), one in Italy (58), and one in Germany (59). Chronic musculoskeletal pain addressed in these studies included osteoarthritis (OA) (*n* = 13), LBP (*n* = 13), fibromyalgia (FM) (*n* = 5), chronic multisite pain in older adults (*n* = 2), non-specific neck pain (*n* = 2), partial anterior cruciate ligament (ACL) injury (*n* = 1), shoulder, neck and back pain (*n* = 1), and rheumatoid arthritis (RA) (*n* = 1). Detailed characteristics of individual studies are presented in Table 1.

**Table 1:** Characteristics of included studies.

First author, published year, country	Chronic musculoskeletal pain	Sample size, mean age (years)	Intervention regimen (Tai Chi type, forms, and duration)	Follow-up (weeks)	Pain intensity assessment	Adverse events	Control group
Adler, 2007, USA (41)	OA	14; 71.60 ± 6.80	Wu Tai Chi; 16-forms; 10 weeks	–	WOMAC pain	No report	Bingo activity
Brisme'e, 2007, USA (42)	KOA	31; 70.00 ± 9.20	Yang Tai Chi; 24-forms; 12 weeks	6	VAS pain; WOMAC pain	Minor muscle soreness and foot and knee pain, not numbers reported	Health education
Fransen, 2007, Australia (55)	Hip or knee OA	97; 70.29 ± 6.21	Sun Tai Chi; 24-forms; 12 weeks	12	WOMAC pain	Exacerbation of LBP (*n* = 1)	Waiting list
Hartman, 2000, USA (43)	Lower extremity OA	33; 68	Yang Tai Chi; 9-forms; 12 weeks	–	ASES pain; AIMS pain	No report	Routine care
Hu, 2019, China (22)	KOA	92; 65.98 ± 3.92	Tai Chi; NR; 24 weeks	–	VAS pain; WOMAC pain	No report	Health education
Kang, 2022, China (23)	KOA	27; 64.10 ± 5.40	Tai Chi; NR; 36 weeks	–	WOMAC pain	No report	Health education
Lee, 2018, USA (44)	KOA	182; 61 ± 10.00	Yang Tai Chi; 10-forms; 12 weeks	–	WOMAC pain	No report	Physical therapy
Song, 2007, Korea (53)	OA	43; 63	Sun Tai Chi; 12-forms; 12 weeks	–	WOMAC pain	No report	No treatment
Song, 2022, China (24)	KOA	40; 64.15 ± 8.56	Yang Tai Chi, 8-forms; 12 weeks	24	WOMAC pain	No adverse events	Health education
Tsai, 2013, USA (45)	KOA	55; 78.91	Sun Tai Chi; 12-forms; 20 weeks	–	WOMAC pain	No adverse events	Health education
Wang, 2009, USA (46)	KOA	40; 65	Yang Tai Chi, 10-forms; 12 weeks	48	WOMAC pain	Knee pain, resolved after modification of the participant's Tai Chi technique (*n* = 1)	Health education plus stretching program
Wang, 2016, USA (47)	KOA	204; 60.20 ± 10.47	Yang Tai Chi; NR; 12 weeks	52	WOMAC pain	No adverse events	Physical therapy
Wang, 2021, China (25)	KOA	165; 56.99 ± 6.52	Tai Chi, 24-forms; 12 weeks	–	VAS pain	No report	Baduanjin, Wuqinxi
Chang, 2024, China (26)	LBP	108; 38.12 ± 11.53	Tai Chi; 8-forms; 12 weeks	12	NRS pain	LBP-related pain (*n* = 4)	Health education
Chen, 2023, China (27)	LBP	74; 68.04 ± 8.62	Ai-Chi; 16-forms; 8 weeks	–	VAS pain	No report	Core stability training
Hall, 2011, Australia (56)	LBP	160; 44.40 ± 13.20	Tai Chi; NR; 10 weeks	–	NRS pain	Back pain, relieved by correction of upper limb posture (*n* = 4)	Waiting list
Liu, 2019, China (28)	LBP	43; 59.38 ± 4.26	Chen Tai Chi; 16-forms; 12 weeks	–	VAS pain	No report	Core stabilization exercise; no treatment
Lu, 2017, China (29)	LBP	108; 62.67 ± 6.31	Tai Chi; 24-forms; 12 weeks	12	VAS pain; SF-36 BP	No report	Celecoxib
Tong, 2016, China (30)	LBP	64; 32.63 ± 6.44	Tai Chi “pushing hand”; 6-forms; 4 weeks	–	VAS pain; ODI pain	No report	No treatment
Tong, 2017, China (31)	LBP	71; 41.95 ± 4.16	Tai Chi “flash back”; NR; 12 weeks	12	VAS pain	No report	Core stabilization exercise
Wang, 2020, China (32)	LBP	45; 32.89 ± 9.91	Ai-Chi; NR; 6 weeks	6	NRS pain	No report	Core stability training; physical therapy
Wang, 2021, China (33)	LBP	20; 63.70 ± 3.80	Tai Chi; 8-forms; 12 weeks	–	NRS pain	No adverse events	Physical therapy
Wang, 2024, China (34)	LBP	109; 36.55 ± 13.00	Tai Chi; 8-forms; 12 weeks	24	NRS pain	Exacerbation of LBP (*n* = 6), other pain (*n* = 3)	Health education and Physical therapy
Wu, 2013, China (35)	LBP	320; 37.60 ± 5.40	Chen Tai Chi; 24-forms; 24 weeks	–	VAS pain	No report	Backward walking; jogging; swimming; no exercise
Yan, 2022, China (36)	LBP	20; 69.00 ± 1.56	Tai Chi; 24-forms; 6 weeks	–	VAS pain	No report	No treatment
Zeng, 2021, China (37)	LBP	39; 29.93 ± 9.72	Ai-Chi; 19-forms; 6 weeks	–	VAS pain; RMDQ pain	No report	Core stability training
Bongi, 2016, Italy (58)	FM	44; 52.24 ± 12.19	Tai Chi; NR; 16 weeks	–	SF-36 BP; WPI pain	No report	Health education
Jones, 2012, USA (48)	FM	98; 54	Yang Tai Chi; 8-forms; 12 weeks	–	BPI pain; FIQ pain	No report	Health education
Wang, 2010, USA (49)	FM	66; 50.10 ± 11.10	Yang Tai Chi; 10-forms; 12 weeks	12	FIQ pain; VAS pain	No adverse events	Health education plus stretching
Wang, 2018, USA (50)	FM	226; 52 ± 11.95	Yang Tai Chi; NR; 24 weeks	28	FIQ pain	Minor musculoskeletal events (*n* = 8)	Aerobic exercise
Wong, 2018, Korea (54)	FM	31; 51.00 ± 2.00	Yang Tai Chi; NR; 12 weeks		VAS pain	No report	No treatment
Büyükturan, 2019, Turkey (57)	Partial ACL injury	58; 25.50 ± 6.40	Yang Tai Chi; 10-forms; 24 weeks	–	VAS pain	No report	No treatment
Lauche, 2016, Germany (59)	Non-specific neck pain	114; 49.40 ± 11.70	Yang Tai Chi; 13-forms; 12 weeks	12	VAS pain; SF-36 BP	Achilles tendon pain (*n* = 2), and migraine (*n* = 1)	Neck exercise; waiting list
Zou, 2024, China (38)	Non-specific neck pain	35; 51.31 ± 3.48	Chen Tai Chi; 18-forms; 12 weeks		VAS pain; SF-36 BP	No report	Health education
Mao, 2021, China (39)	Chronic multisite pain	67; 63.96 ± 6.99	Tai Chi; 24-forms; 8 weeks	24	VAS pain; CPSS pain	No report	No treatment
Wang, 2008, USA (51)	RA	20; 49.50 ± 13.66	Yang Tai Chi; NR; 12 weeks	12	VAS pain; SF-36 BP	No adverse events	Health education plus stretching
Xu, 2019, China (40)	Shoulder, neck, back pain	350; 21.20 ± 1.67	Chen Tai Chi; 16-forms; 12 weeks	–	VAS pain	No report	No treatment
You, 2018, USA (52)	Chronic multisite pain	45; 74.53 ± 7.24	Yang Tai Chi; 8-forms; 12 weeks	24	BPI pain	No report	Light physical exercise

ACL, anterior cruciate ligament; ACR, American College of Rheumatology; AIMS, arthritis impact measurement scale; ASES, arthritis self-efficacy scale; BPI, brief pain inventory; CPSS, chronic pain self-efficacy scale; FM, fibromyalgia; FIQ, fibromyalgia impact questionnaire; KOA, knee osteoarthritis; LBP, low back pain; NRS, numerical rating scale; OA, osteoarthritis; ODI, Oswestry disability index; RA, rheumatoid arthritis; RMDQ, Roland Morris disability questionnaire; SF-36 BP, survey short form 36 (SF-36) bodily pain; VAS, visual analogue scale; WOMAC, Western Ontario and McMaster Universities osteoarthritis index; WPI, widespread pain index.

### 3.2 Assessment of risk of bias

Randomization process: Of the included studies, 25 studies (22, 25, 27, 28, 30–33, 35, 36, 39–42, 44, 45, 48, 49, 51–55, 57, 58) failed to provide detailed descriptions of allocation concealment methods, rating as “some concerns”. One study (43) was rated as “high risk” due to the baseline differences between the intervention group and the control group, while the remaining 12 studies (23, 24, 26, 29, 34, 37, 38, 46, 47, 50, 56, 59) were rated as “low risk”, with a clearly described randomization procedure and allocation concealment.

Deviations from intended interventions: Due to the inherent challenges of blinding in Tai Chi, interventions were generally unable to maintain blinding of participants and personnel, resulting in a rating of “some concerns”.

Missing outcome data: Two studies (34, 53) were rated as “high risk” due to high dropout rates (43% in the Tai Chi group and 39% in the control group) without appropriate handling of missing data. And other studies were rated as “low risk”.

Measurement of the outcome: Although some studies reported blinding of outcome assessors, this was considered insufficient to mitigate detection bias because pain was a patient-reported outcome and participants were not blinded. Accordingly, all studies were rated as having some concerns regarding the measurement of the outcome.

Selection of the reported result: 20 studies (24, 26, 28, 29, 33, 34, 39, 41, 44–50, 53–56, 59) were rated as “low risk” as they were prospectively registered and adhered to the registered protocol. The remaining 18 studies (22, 23, 25, 27, 30–32, 35–38, 40, 42, 43, 51, 52, 57, 58) lacked sufficient details to verify protocol adherence and were rated as “some concerns”.

**Figure 2:**
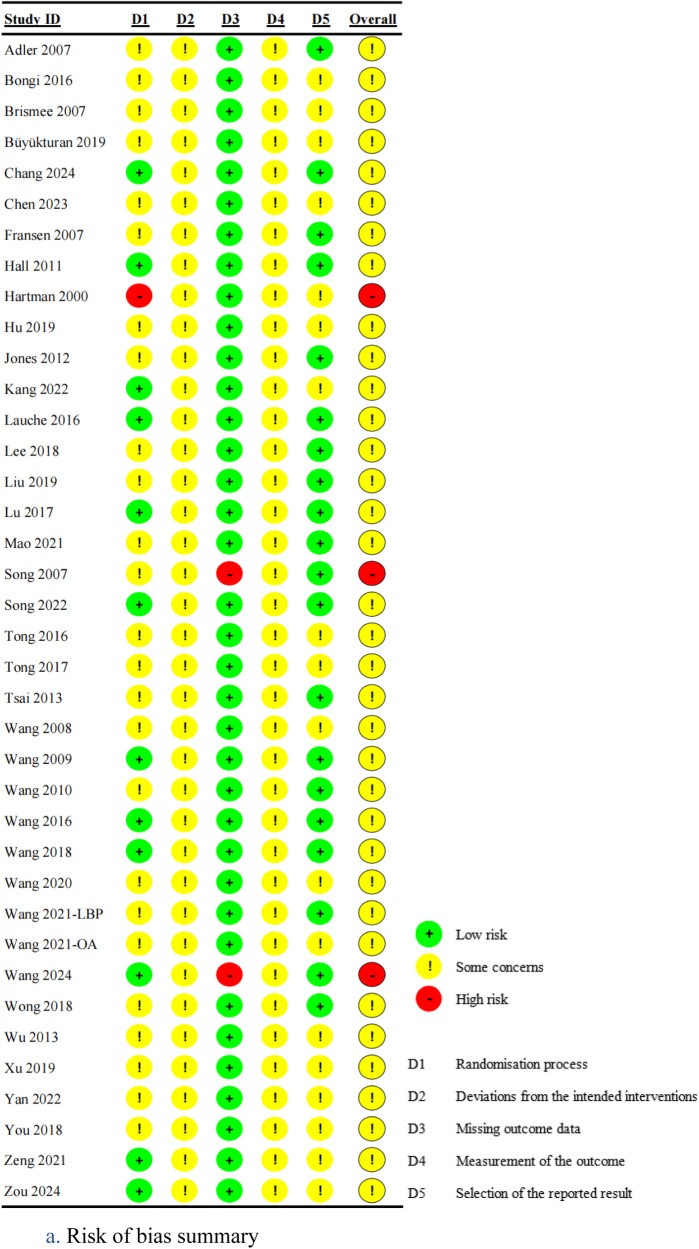
**(a)** risk of bias summary. **(b)** Risk of bias graph.

Overall bias: Three studies (34, 43, 53) were rated as “high risk” due to deficiencies in either the randomization process or missing outcome data domains. The remaining 35 studies were rated as “some concerns” due to varying degrees of methodological limitations across domains. Detailed results are presented in Figures 2a,b.

**Figure:**
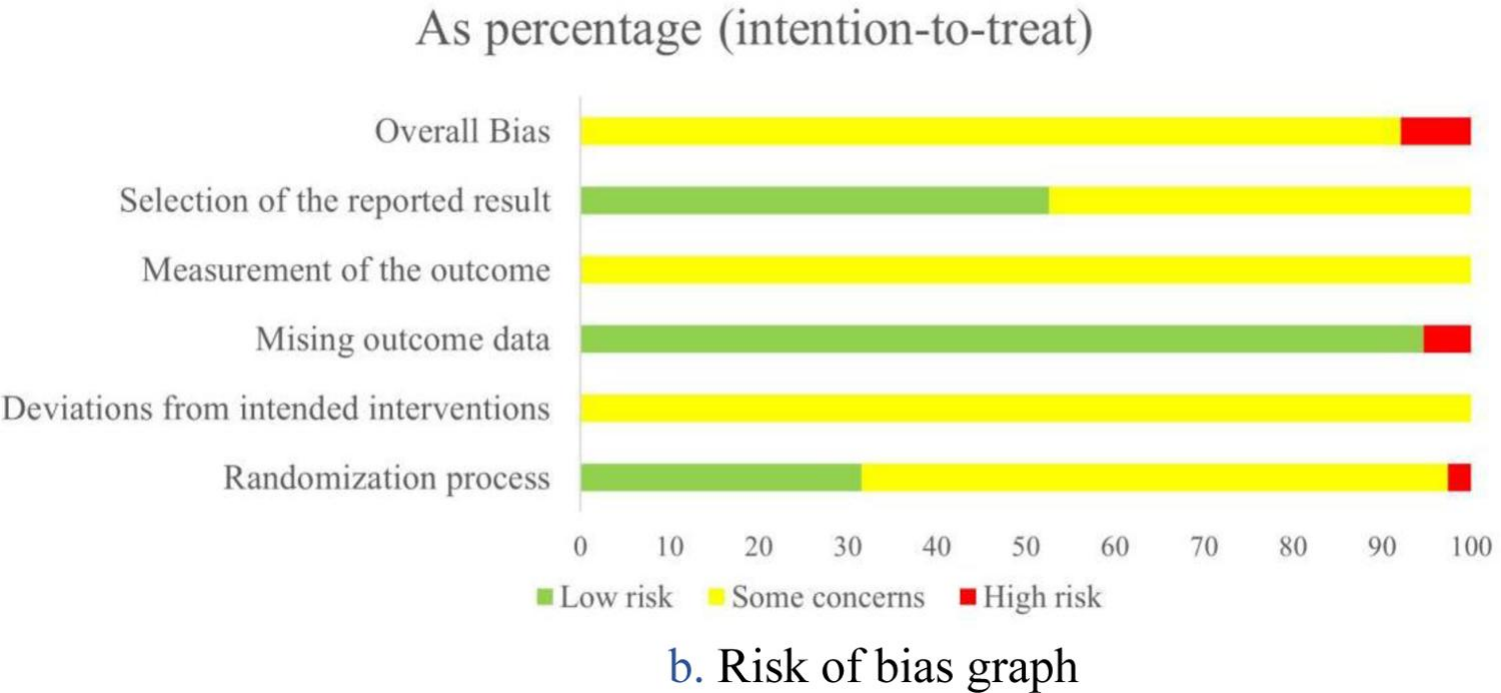


### 3.3 Primary outcome: efficacy of Tai Chi for chronic musculoskeletal pain

#### 3.3.1 Overall pooled effect

**Figure 3:**
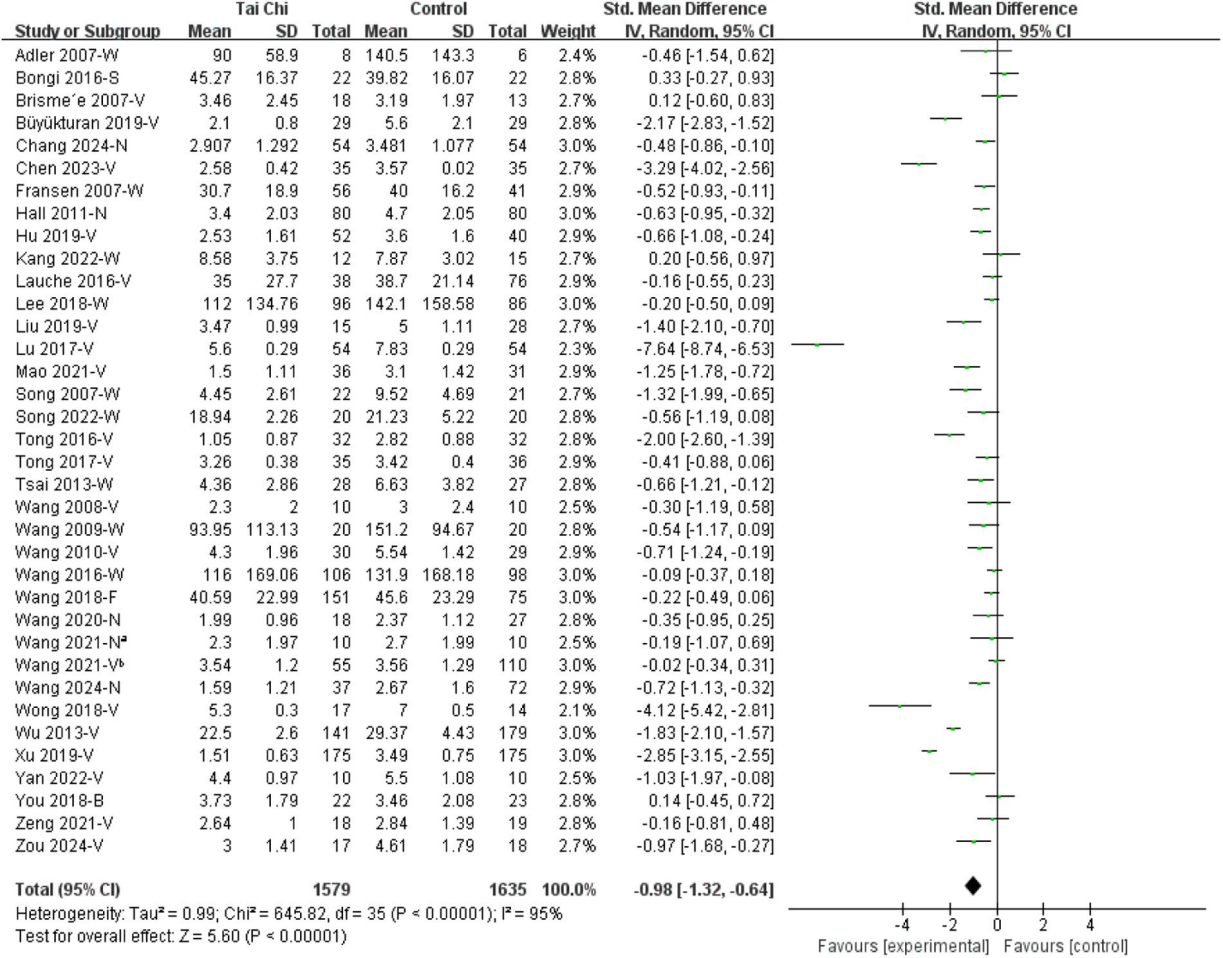
Forest plots of Tai Chi on chronic musculoskeletal pain. B, brief pain inventory; F, fibromyalgia impact questionnaire; N, numerical rating scale; S, survey short form 36 bodily pain; V, visual analogue scale; W, Western Ontario and McMaster Universities osteoarthritis index. Wang 2021-N^a^ [Ref. (33)] and Wang 2021-V^b^ [Ref. (25)] denote two separate studies.

Of all included studies, 36 studies were eligible for meta-analysis, while two studies (43, 48) were excluded due to insufficient data. The findings indicated that Tai Chi effectively managed chronic musculoskeletal pain when compared to control groups, with an SMD of −0.98 [95% CI (−1.32, −0.64)] (Figure 3). However, given the high heterogeneity among the included studies (*I*^2^ = 95%, *p* < 0.01), subgroup analyses were conducted based on the disease category and control type to explore potential sources of variability.

#### 3.3.2 Subgroup analysis by disease category

##### 3.3.2.1 Tai Chi for OA

**Figure 4:**
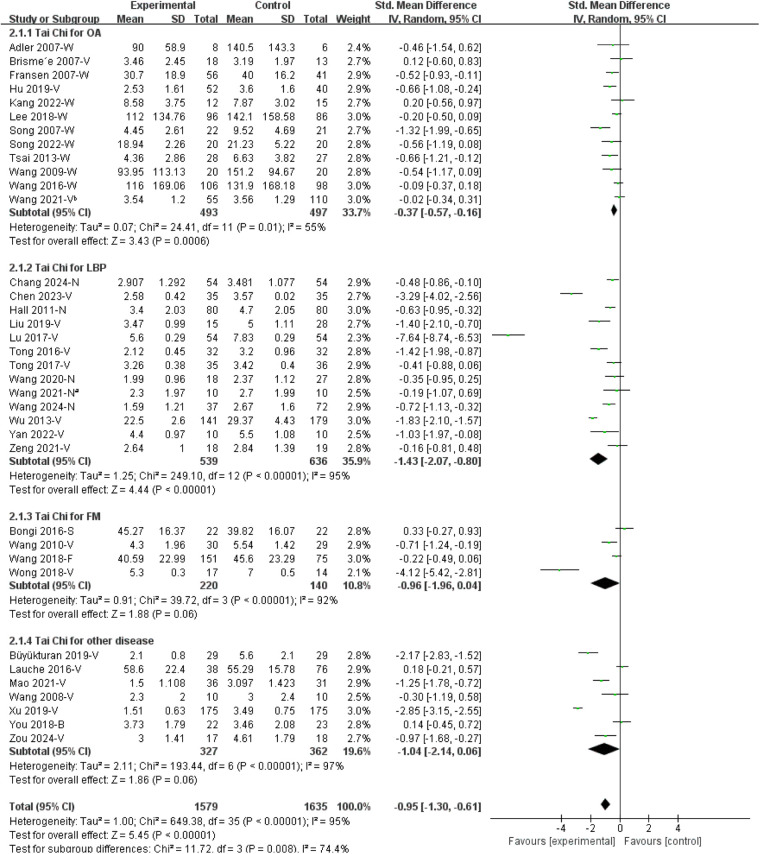
Subgroup analysis of Tai Chi on chronic musculoskeletal pain by disease category. B, Brief Pain Inventory; FM, fibromyalgia; F, fibromyalgia impact questionnaire; LBP, low back pain; N, numerical rating scale; OA, osteoarthritis; S, survey short form 36 bodily pain; V, visual analogue scale; W, Western Ontario and McMaster Universities osteoarthritis index. Wang 2021-N^a^ [Ref. (33)] and Wang 2021-V^b^ [Ref. (25)] denote two separate studies.

Twelve studies (22–25, 41, 42, 44–47, 53, 55) reported the effects of Tai Chi for OA. The combined findings suggested that Tai Chi was associated with a modest pain reduction among OA patients compared to control groups, with an SMD of −0.37 [95% CI (−0.57, −0.16)] (Figure 4).

##### 3.3.2.2 Tai Chi for LBP

Thirteen studies (26–37, 56) evaluated the effects of Tai Chi on LBP. Pooled analysis indicated an association between Tai Chi and reduced pain intensity compared with control interventions, with an SMD of −1.43 [95% CI (−2.07, −0.80)] (Figure 4).

##### 3.3.2.3 Tai Chi for FM

Four studies (49, 50, 54, 58) investigated the effects of Tai Chi on FM. The combined results revealed a trend toward pain reduction on Tai Chi with control interventions, showing an SMD of −0.96 [95% CI (−1.96, 0.04)]; however, the effect did not reach statistical significance (Figure 4).

##### 3.3.2.4 Tai Chi for other diseases

Seven studies (38–40, 51, 52, 57, 59) investigated the therapeutic effects of Tai Chi on various conditions, including partial ACL injury (57), nonspecific neck pain (38, 59), RA (51), shoulder, neck and back pain (40), and chronic multisite pain in older adults (39, 52). The pooled analysis showed a point estimate favouring Tai Chi, with an SMD of −1.04 [95% CI (−2.14, 0.06)] for chronic musculoskeletal pain across these conditions; however, the effect did not reach statistical significance (Figure 4).

#### 3.3.3 Subgroup analysis by control type

**Figure 5:**
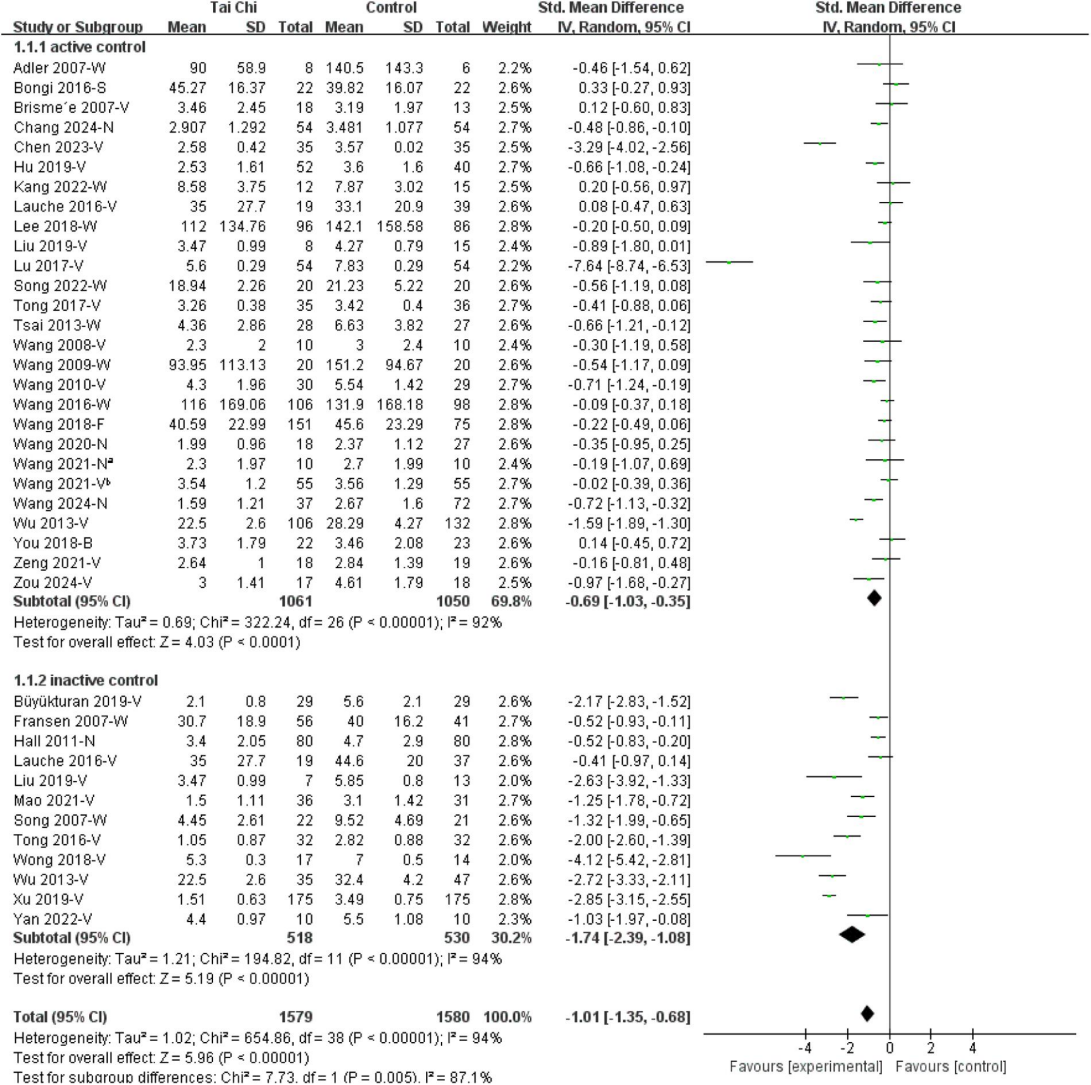
Subgroup analysis of Tai Chi on chronic musculoskeletal pain by control type. B, brief pain inventory; FM, fibromyalgia; F, fibromyalgia impact questionnaire; LBP, low back pain; N, numerical rating scale; OA, osteoarthritis; S, survey short form 36 bodily pain; V, visual analogue scale; W, Western Ontario and McMaster Universities osteoarthritis index. Active control, Aerobic exercise, Baduanjin, backward walking, bingo activity, core stabilization, celecoxib, health education, jogging, light physical exercise, physical therapy, stretching program, swimming, Wuqinxi; Inactive control group = waiting list, routine care, no treatment, no exercise. Wang 2021-N^a^ [Ref. (33)] and Wang 2021-V^b^ [Ref. (25)] denote two separate studies.

When stratified by control type, Tai Chi demonstrated a statistically significant reduction in pain compared with both active controls SMD of −0.69 [95% CI (−1.03, −0.35)] and inactive controls SMD of −1.74 [95% CI (−2.39, −1.08)]. The magnitude of effect appeared larger when Tai Chi was compared with inactive controls; however, substantial heterogeneity was observed in both subgroups, and the between-subgroup difference should be interpreted with caution (Figure 5).

### 3.4 Publication bias

**Figure 6:**
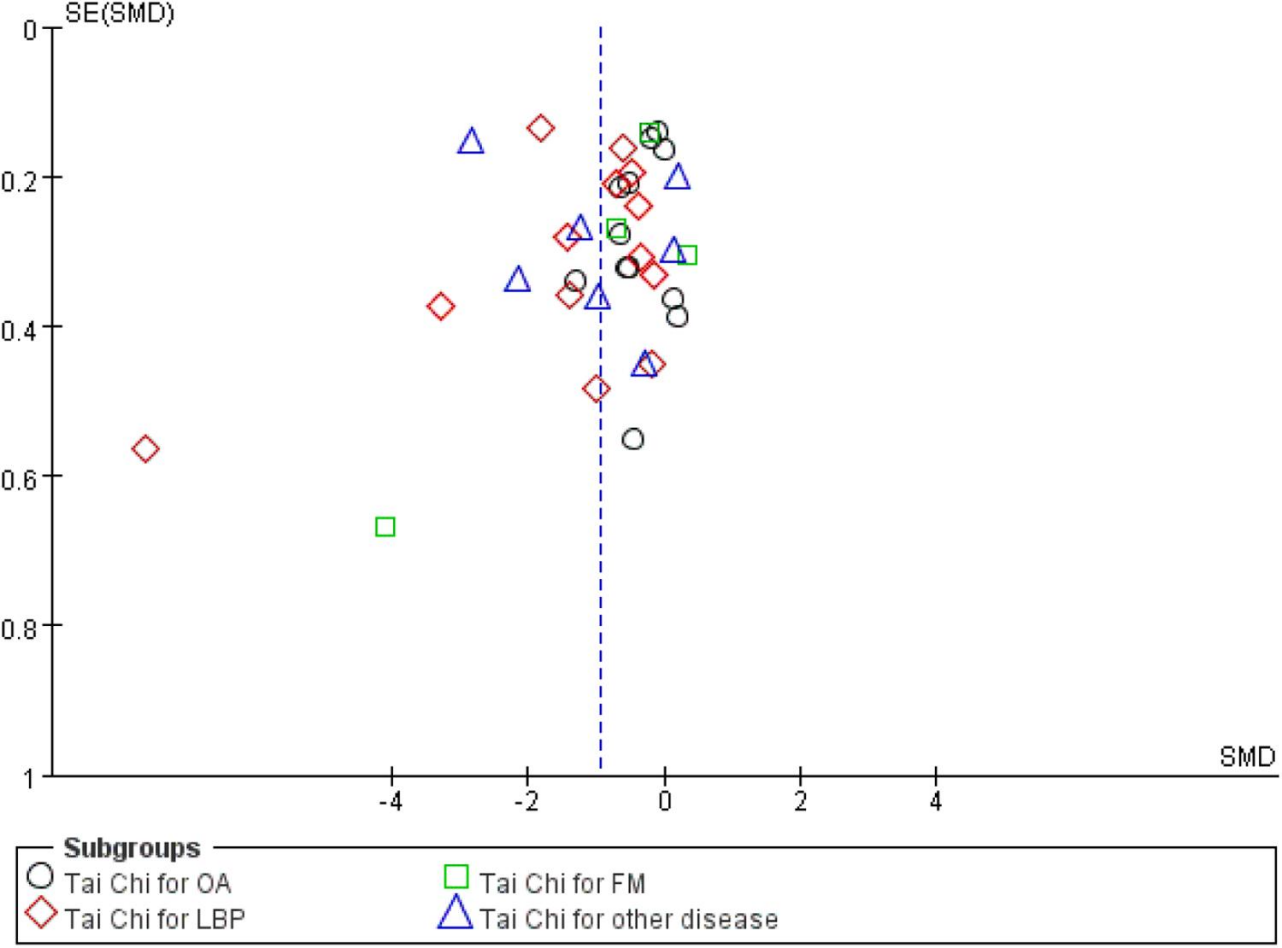
Funnel plot for included studies. FM, fibromyalgia; LBP, low back pain; OA, osteoarthritis.

As shown in Figure 6, the funnel plot analysis suggested no significant evidence of publication biases among the included studies. This observation was corroborated by the results of both Begg's and Egger's tests, which also showed no statistically significant biases (*p* > 0.05).

### 3.5 Secondary outcomes: adverse events

Among the 28 studies reviewed, comprising 74% of the total, it was observed that certified Tai Chi instructors possessed a solid foundation in Tai Chi and had undergone rigorous training to ensure accurate execution of movements. Nonetheless, 10 studies (26, 30, 34, 35, 37, 41, 42, 51, 55, 58) did not furnish information regarding the qualifications of the instructors.

Minor AEs attributed to Tai Chi exposure were observed during the practice period, encompassing minor musculoskeletal discomfort and a singular instance of a migraine attack.

In Hall's study (56), three participants noted a transient increase in back pain symptoms, which subsided by the third or fourth week of treatment, and one individual reported improved upper back pain upon correction of upper extremity posture. Another study reported increased knee pain, which was alleviated by modifying the Tai Chi technique (46). Additionally, one participant experienced exacerbation of chronic back pain, leading to withdrawal from the study (55). Wang's remote Tai Chi intervention was limited by reduced precision in movement correction, and both intervention and control groups experienced increased waist and knee pain due to non-standard movements (34). The frequency and probability of AEs are presented in Table 2. However, one study (24) has revealed that muscle soreness after Tai Chi practice should not be classified as an AE, given the nature of the exercise.

**Table 2:** Adverse events reporting form.

First author, published year, country	AEs in Tai Chi group	Total	Incidence rates	AEs in control group	Total	Incidence rates
Brisme'e, 2007, USA	Some	18	–	0	13	0.00%
Chang, 2024, China	4	46	8.70%	0	41	0.00%
Fransen, 2007, Australia	1	56	1.79%	1	41	2.44%
Hall, 2011, Australia	4	80	5.00%	0	80	0.00%
Lauche, 2016, Germany	3	38	7.89%	1	76	1.32%
Wang, 2009, USA	1	20	5.00%	0	20	0.00%
Wang, 2018, USA	8	151	5.30%	4	75	5.33%
Wang, 2024, China	9	37	24.32%	13	72	18.06%

AE, adverse events.

### 3.6 Exploratory subgroup analyses of intervention characteristics

Given the substantial heterogeneity observed in the primary meta-analyses, exploratory subgroup analyses were conducted to examine whether intervention characteristics, including intervention duration, Tai Chi styles and Tai Chi forms, might modify the treatment effects. These analyses were prespecified as exploratory effect modifier analyses rather than outcomes and were performed to aid interpretation of heterogeneity rather than to draw definitive conclusions.

#### 3.6.1 Intervention durations

Exploratory subgroup analyses by intervention durations showed varying effect estimates across duration categories (12 weeks, less than 12 weeks, and more than 12 weeks); however, heterogeneity remained substantial within subgroups [*I*^2^ = 89%–96%], and no significant between-subgroup difference was observed (*p* for subgrou*p* = 0.68), precluding no clear dose–response relationship between intervention duration and pain reduction was observed (Supplementary Figure S1).

#### 3.6.2 Tai Chi types

Exploratory subgroup analyses by Tai Chi type did not demonstrate statistically significant differences between subgroups (*p* for subgroup = 0.16); moreover, substantial heterogeneity persisted within several subgroups, limiting definitive interpretation (Supplementary Figure S2).

#### 3.6.3 Tai Chi forms

Exploratory subgroup analyses based on the number of Tai Chi forms showed variable effect estimates across form categories. Some subgroups (6-forms, 12-forms, 16-forms, and 24-forms Tai Chi) showed pooled effect estimates favoring Tai Chi; however, substantial heterogeneity was observed within most subgroups, and several subgroups were informed by only one or two trials. These limitations preclude definitive conclusions regarding an optimal number of Tai Chi forms (Supplementary Figure S3).

### 3.7 Certainty of evidence (GRADE)

Certainty of evidence for the primary outcomes was evaluated as low to very low. The certainty of the evidence for the primary outcomes was predominantly downgraded due to inconsistencies in measurement tools across studies and different conditions. Table 3 provides a detailed breakdown of the certainty surrounding the primary outcomes.

**Table 3:** Quality of evidence of primary outcomes.

Outcomes	Study design	Risk of bias	Inconsistency	Indirectness	Imprecision	Publication bias	Large effect	Plausible confounding	Dose response gradient	Number of patients		Certainty
										Exp	Con	
OA	RCTs	Serious^a^	Serious^c^	Not serious	Not serious	Undetected	NA	NA	NA	511	512	Low
LBP	RCTs	Serious^a^	Serious^b^	Not serious	Not serious	Undetected	NA	NA	NA	539	636	Low
FM	RCTs	Serious^a^	Serious^b^	Not serious	Serious^d^	Undetected	NA	NA	NA	271	187	Very low
Other	RCTs	Serious^a^	Serious^b^	Not serious	Serious^d^	Undetected	NA	NA	NA	327	362	Very low

FM, fibromyalgia; LBP, low back pain; NA, not applicable; OA, osteoarthritis; other, partial anterior cruciate ligament injury, non-specific neck pain, rheumatoid arthritis, shoulder neck and back pain, and chronic multisite pain.

High quality: Further research is very unlikely to change our confidence in the estimate of effect; Moderate quality: Further research is likely to have an important impact on our confidence in the estimate of effect and may change the estimate; Low quality: Further research is very likely to have an important impact on our confidence in the estimate of effect and is likely to change the estimate; Very low quality: The results may vary significantly from the true values, and further research is highly likely to alter the outcomes.

^a^ Risk of bias downgraded due to high or unclear risk in key domains across most studies.

^b^ Inconsistency downgraded due to substantial heterogeneity across studies with considerable variation in effect sizes.

^c^ Inconsistency downgraded due to moderate heterogeneity and variation in effect sizes.

^d^ Imprecision downgraded due to small total sample size.

## 4 Discussion

### 4.1 Summary of the main findings

This review suggests that Tai Chi was associated with pain reduction compared with the control groups in OA and LBP, whereas evidence for FM and other diseases remains inconclusive. In addition, no serious AEs related to Tai Chi were reported, which is consistent with previous evidence supporting the safety of Tai Chi practice (60). Nevertheless, as Tai Chi is a physical exercise intervention, monitoring exercise intensity remains advisable (61), particularly in populations with chronic musculoskeletal pain.

Despite these findings, the reliability and generalizability of results are limited by heterogeneity in interventions and controls, lack of direct comparisons for some therapies, and a small number of studies for certain interventions. This likely reflects real-world variability in Tai Chi interventions and study populations rather than a single identifiable source. Consequently, the pooled estimates represent an average effect across heterogeneous contexts and should not be interpreted as precise estimates for any specific Tai Chi regimen or patient group. Furthermore, while standardized mean differences allowed pooling across different pain scales, the minimum clinically important difference could not be applied due to variations in scales, comparators, and reporting standards. We highlight the minimum clinically important difference as an important consideration for future research to enhance the clinical interpretability of Tai Chi's efficacy.

### 4.2 Comparison with previous review

Prior to this review, four related systematic reviews have drawn similar conclusions regarding the favorable effects of Tai Chi on various chronic conditions. These reviews highlighted the positive impact of Tai Chi on OA (14–16), LBP (14–17), osteoporosis (15), and headache (16). However, the evidence regarding the effectiveness of Tai Chi for FM pain showed discrepancies between studies conducted by Kong and Peng. Peng's study reported significant improvements in FM pain with Tai Chi (14), contrasting with Kong's findings (15). In our review, pooling data from four RCTs did not allow firm conclusions to be drawn regarding the association between Tai Chi and FM pain. Therefore, further high-quality, large-sample RCTs are necessary to solidify this evidence.

Notably, previous reviews have rarely examined intervention characteristics of Tai Chi in detail. In our study, intervention durations, Tai Chi types, and number of Tai Chi forms were explored using prespecified exploratory subgroup analyses to assess whether these characteristics might partly contribute to the observed heterogeneity in treatment effects. Across included trials, intervention durations varied, with 12 weeks being the most commonly reported; however, subgroup analyses showed substantial within-group heterogeneity and no significant between-group differences, precluding any inference regarding an optimal duration or dose–response relationship. Similarly, effect estimates varied across Tai Chi styles and forms, but these findings were characterized by considerable heterogeneity and were often informed by a limited number of studies. Taken together, these exploratory analyses should be interpreted cautiously and are intended to generate hypotheses rather than to support regimen-specific recommendations. Further well-designed trials with standardized intervention reporting are needed to clarify whether specific Tai Chi characteristics meaningfully influence analgesic outcomes.

Another notable distinction is the inclusion of safety data in our review, which was lacking in two previous reviews (14, 16). While Tai Chi appears to be a generally safe intervention with no serious AEs reported, cautious interpretation is warranted given the limited and heterogeneous reporting of AEs across trials. By jointly considering efficacy and safety, this review provides an updated and integrative synthesis of the available evidence on Tai Chi for chronic musculoskeletal pain, while highlighting important uncertainties that warrant further high-quality research.

### 4.3 Implications for future practice and research

In clinical settings, Tai Chi may represent a safe and potentially beneficial option for managing chronic musculoskeletal pain. Exploratory analyses suggested that intervention characteristics such as type of Tai Chi and number of forms may influence treatment effects; however, these findings should be interpreted cautiously, given the substantial heterogeneity and limited number of trials within subgroups. Accordingly, no specific Tai Chi regimen can be recommended at this stage. Future investigations could broaden their scope to include assessments of physical function and mental well-being alongside pain, addressing the scarcity of interventions targeting symptom clusters. Studies focusing on Tai Chi's effects on pain should incorporate both subjective and objective measures to ensure robustness in chronic musculoskeletal pain assessment. Moreover, our review identified several areas for potential improvement. Among the included RCTs, a proportion of studies did not report documented trial registration. Compliance with registration requirements outlined by the International Committee of Medical Journal Editors and the WHO (62, 63) is crucial to enhance transparency, mitigate publication bias, prevent redundant research efforts, and ensure updated RCT information. To uphold higher standards of evidence, reviewers are strongly encouraged to proactively register trials, with the International Clinical Trials Registry Platform being a recommended open-access database.

Lastly, given Tai Chi's acceptability and potential contribution to chronic musculoskeletal pain management, this cost-effective and self-practice therapy could lead to reduced healthcare expenditures and societal burdens. Future research should incorporate economic data collection to better inform its potential role in sustainable pain management strategies.

### 4.4 Strengths and limitations

A key strength of this review is its comprehensive search strategy, which covered eight electronic databases and two trial registration platforms, including major English- and Chinese-language sources, to achieve broad coverage of the available literature. Nevertheless, several limitations should be acknowledged. Substantial unexplained heterogeneity, limited blinding in most trials, variability in comparator interventions, and generally small sample sizes may have introduced bias and reduced the certainty and clinical interpretability of the pooled estimates. In addition, the exclusion of studies published in languages other than English and Chinese may have resulted in the omission of relevant evidence. Finally, the findings are primarily applicable to OA and LBP and may not be generalisable to other musculoskeletal pain conditions.

## 5 Conclusion

Current evidence suggests that Tai Chi may reduce pain in OA and LBP, supporting its role as a potentially beneficial intervention for these conditions. However, due to methodological limitations and low certainty of evidence, these findings should be interpreted cautiously. More rigorously designed and registered trials with long-term safety evaluation are needed to confirm its clinical value.

## Data Availability

The original contributions presented in the study are included in the article/Supplementary Material, further inquiries can be directed to the corresponding author.
