# Supplementary material for: Efficacy and safety of Tai Chi for chronic musculoskeletal pain: a systematic review and meta-analysis
Source: Front Pain Res (Lausanne). 2026 Feb 24;7:1678660. doi: 10.3389/fpain.2026.1678660 (PMC12971446; doi:10.3389/fpain.2026.1678660)
Supplement: Supplementary file 1 [file Table1.docx]

**Supplementary data**

Appendix 1: Search strategy and results

| Section | Purpose | Time | Content |
| --- | --- | --- | --- |
| Appendix 1A | Initial database search | Database inception – May 2025 | Chronic pain-based strategy |
| Appendix 1B | Supplementary search | Database inception – January 2026 | Chronic musculoskeletal pain-based strategy |

Table S1A. Original search strategy and results (May 2025)

| **Data Sources** | | **Search Terms and Combinations** | **Records retrieved (n)** |
| --- | --- | --- | --- |
| AMED | #1 Pain [All Text]  #2 Chronic pain [All Text]  #3 #1 OR #2  #4 Tai Ji [All Text]  #5 Tai Ji Quan [All Text]  #6 Tai Chi [All Text]  #7 Tai Chi Chuan [All Text]  #8 Mind-body therapy [All Text]  #9 Mind-body exercise [All Text]  #10 #4 OR #5 OR #6 OR #7 OR #8 OR #9  #11 #3 AND #10 | | n=81 |
| MEDLINE | #1 (MH “Pain”)  #2 Pain [Title/Abstract]  #3 (MH “Chronic Pain”)  #4 Chronic Pain [Title/Abstract]  #5 #1 OR #2 OR #3 OR #4  #6 (MH “Tai Ji”)  #7 Tai Ji [Title/Abstract]  #8 Tai Ji Quan [Title/Abstract]  #9 Tai Chi [Title/Abstract]  #10 Tai Chi Chuan [Title/Abstract]  #11 Mind-body exercise [Title/Abstract]  #12 Mind-body therapy [Title/Abstract]  #13 #6 OR #7 OR #8 OR #9 OR #10 OR #11 OR #12  #14 #5 AND #13 | | n=795 |
| EMBASE | #1 ‘pain’/exp OR pain  #2 ‘chronic pain’/exp OR ‘chronic pain’  #3 #1 OR #2  #4 ‘tai ji’/exp OR ‘tai ji’  #5 ‘tai ji quan’ OR ‘tai chi’ OR ‘tai chi chuan’ OR ‘mind-body therapy’ OR ‘mind-body exercise’  #6 #4 OR #5  #7 #3 AND #6 | | n=1408 |
| Web of Science | #1 Pain (Topic)  #2 Chronic pain (Topic)  #3 Tai Ji (Topic)  #4 Tai Ji Quan (Topic)  #5 Tai Chi (Topic)  #6 Tai Chi Chuan (Topic)  #7 Mind-body therapy (Topic)  #8 Mind-body exercise (Topic)  #9 #1 OR #2  #10 #3 OR #4 OR #5 OR #6 OR #7 OR #8  #11 #9 AND #10 | | n=1105 |
| CBM  (Chinese) | #1 “疼痛” [不加权:扩展]  #2 “慢性疼痛” [常用字段:智能]  #3 (#1) OR (#2)  #4 “太极拳” [不加权: 扩展]  #5 “太极” [常用字段:智能] OR “身心疗法” [常用字段: 智能] OR “身心运动” [常用字段: 智能]  #6 (#4) OR (#5)  #7 ((#6) AND (#3)) AND (随机对照试验 [文献类型]) AND (人类 [特征词]) | | n=327 |
| CNKI  (Chinese) | #1 主题=疼痛 OR 慢性疼痛 OR 痛  #2 主题=太极 OR 太极拳 OR 身心疗法 OR 身心运动  #3 摘要=随机 OR 随机对照 OR 随机研究  #4 #1 AND #2 AND #3 | | n=407 |
| VIP  (Chinese) | #1 题名或关键词: 疼痛+慢性疼痛+痛  #2 题名或关键词: 太极+太极拳+身心疗法+身心运动  #3 文摘: 随机+随机对照+随机研究  #4 #1 AND #2 AND #3 | | n=197 |
| Wangfang (Chinese) | #1 题名或关键词: (疼痛 OR 慢性疼痛 OR 痛)  #2 题名或关键词: (太极 OR 太极拳 OR 身心疗法 OR身心运动)  #3 摘要: (随机 OR 随机对照 OR 随机研究)  #4 #1 AND #2 AND #3 | | n=435 |

Table S1B. Supplementary chronic musculoskeletal pain search and results (January 2026)

| **Data Sources** | | **Search Terms and Combinations** | **Records retrieved (n)** |
| --- | --- | --- | --- |
| AMED | #1 [All Text] “chronic musculoskeletal pain” OR “musculoskeletal pain” OR “musculoskeletal condition” OR “musculoskeletal disease” OR “musculoskeletal disorder” OR “chronic pain” OR “chronic multisite pain” OR “muscle pain” OR “skeletal muscle pain” OR “skeletal pain” OR “widespread pain” OR “joint pain” OR “back pain” OR “lumbar pain” OR “neck pain” OR “shoulder pain” OR “hip pain” OR “knee pain” OR “osteoarthritis” OR “arthritis” OR “rheumatoid arthritis” OR “fibromyalgia”  #2 [All Text] “tai ji” OR “tai ji quan” OR “tai chi” OR “tai chi chuan” OR “mind-body therapy” OR “mind-body exercise”  #3 #1 AND #2 | | n=69 |
| MEDLINE | #1 MH "musculoskeletal pain+" OR MH "chronic pain" OR MH "pain+" OR MH "neck pain" OR MH "musculoskeletal diseases+" OR MH "back pain" OR MH "shoulder pain" OR MH "low back pain" OR XB **“**chronic musculoskeletal pain” OR XB “chronic multisite pain” OR XB “musculoskeletal pain” OR XB “musculoskeletal disorder” OR XB “musculoskeletal condition” OR XB “musculoskeletal disease” OR XB “muscle pain” OR XB “skeletal muscle pain” OR XB “skeletal pain” OR XB “widespread pain” OR XB “Joint pain” OR XB “Back pain” OR XB “Lumbar pain” OR XB “Neck pain” OR XB “shoulder pain” OR XB “hip pain” OR XB “knee pain” OR XB “osteoarthritis” OR XB “arthritis” OR XB “rheumatoid arthritis” OR XB “fibromyalgia”  #2 [Title/Abstract] “tai ji” OR XB “tai ji quan” OR XB “tai chi” OR XB “tai chi chuan” OR XB “mind-body therapy” OR XB “mind-body exercise”  #3 #1 AND #2 | | n=590 |
| EMBASE | #1 ‘musculoskeletal pain’/exp OR ‘musculoskeletal pain’ OR ‘chronic musculoskeletal pain’ OR ‘chronic multisite pain’ OR ‘musculoskeletal disorder’ OR ‘musculoskeletal condition’ OR ‘musculoskeletal disease’ OR ‘chronic pain’ OR ‘muscle pain’ OR ‘skeletal muscle pain’ OR ‘skeletal pain’ OR ‘widespread pain’ OR ‘joint pain’ OR ‘back pain’ OR ‘lumbar pain’ OR ‘neck pain’ OR ‘shoulder pain’ OR ‘hip pain’ OR ‘knee pain’ OR ‘osteoarthritis’ OR ‘arthritis’ OR ‘rheumatoid arthritis’ OR ‘fibromyalgia’  #2 ‘tai ji’ OR ‘tai ji quan’ OR ‘tai chi’ OR ‘tai chi chuan’ OR ‘mind-body therapy’ OR ‘mind-body exercise’  #3 #1 AND #2 | | n=1375 |
| Web of Science | #1 TX “chronic musculoskeletal pain” OR “musculoskeletal pain” OR “musculoskeletal condition” OR “musculoskeletal disease” OR “musculoskeletal disorder” OR “chronic pain” OR “chronic multisite pain” OR “muscle pain” OR “skeletal muscle pain” OR “skeletal pain” OR “widespread pain” OR “joint pain” OR “back pain” OR “lumbar pain” OR “neck pain” OR “shoulder pain” OR “hip pain” OR “knee pain” OR “osteoarthritis” OR “arthritis” OR “rheumatoid arthritis” OR “fibromyalgia”)  #2 TX (“tai ji” OR “tai ji quan” OR “tai chi” OR “tai chi chuan” OR “mind-body therapy” OR “mind-body exercise”  #3 #1 AND #2 | | n=907 |
| CBM  (Chinese) | #1 肌肉骨骼疼痛'[常用字段: 智能] OR '骨骼痛'[常用字段: 智能] OR '肌肉痛'[常用字段: 智能] OR '腰痛'[常用字段: 智能] OR'背痛'[常用字段: 智能] OR '关节炎'[常用字段: 智能] OR'纤维肌痛症'[常用字段: 智能] OR '疼痛'[常用字段: 智能] OR '痛'[常用字段: 智能]  #3 随机'[常用字段: 智能] OR '随机对照'[常用字段: 智能] OR '随机研究'[常用字段: 智能]  #2 '太极' [常用字段: 智能] OR'身心运动' [常用字段: 智能] OR'身心治疗' [常用字段: 智能]  #3 1 AND #2 | | n=157 |
| CNKI  (Chinese) | #1 主题=肌肉骨骼疼痛 + 骨骼痛 + 肌肉痛 + 腰痛 + 背痛 + 关节炎 + 纤维肌痛症 + 疼痛 + 痛  #2 主题=太极 + 身心运动 + 身心治疗  #3 摘要=随机 + 随机对照 + 随机研究  #4 #1 AND #2 AND #3 | | n=87 |
| VIP  (Chinese) | #1 题名或关键词: 肌肉骨骼疼痛 + 骨骼痛 + 肌肉痛 + 腰痛 + 背痛 + 关节炎 + 纤维肌痛症 + 疼痛 + 痛  #2 题名或关键词: 太极 + 身心运动 + 身心治疗  #3 文摘: 随机 + 随机对照 + 随机研究  #4 #1 AND #2 AND #3 | | n=59 |
| Wangfang (Chinese) | #1 题名或关键词: (肌肉骨骼疼痛 OR 骨骼痛 OR 肌肉痛 OR 腰痛 OR 背痛 OR 关节炎 OR 纤维肌痛症 OR 疼痛 OR 痛)  #2 题名或关键词: (太极 OR 身心运动 OR 身心治疗)  #3 摘要: (随机 OR 随机对照 OR 随机研究)  #4 #1 AND #2 AND #3 | | n=260 |


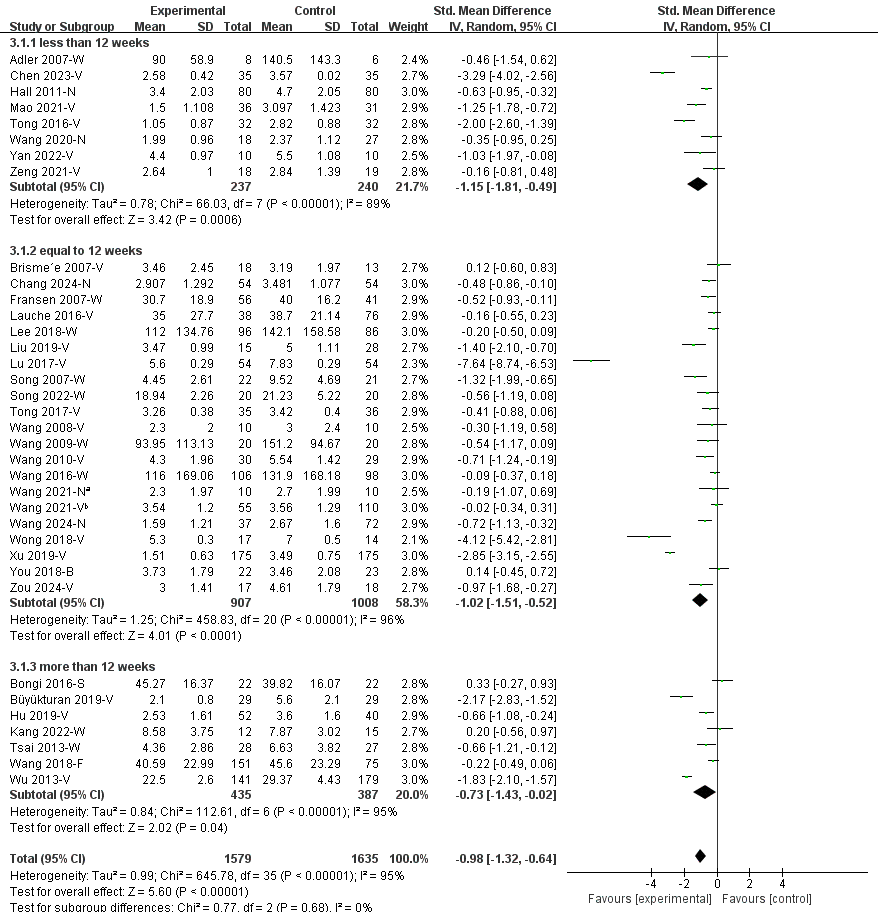


Fig S1. Exploratory subgroup analysis of Tai Chi on chronic musculoskeletal pain by intervention durations. Considerable heterogeneity was observed, and no significant between-subgroup difference was detected.

***Note***: B=Brief Pain Inventory, F=Fibromyalgia Impact Questionnaire, N=Numerical Rating Scale, S=Survey Short Form 36 Bodily Pain, V=Visual Analogue Scale, W=Western Ontario and McMaster Universities Osteoarthritis Index. Wang 2021-N^a^ (Ref. [33]) and Wang 2021-V^b^ (Ref. [25]) denote two separate studies.


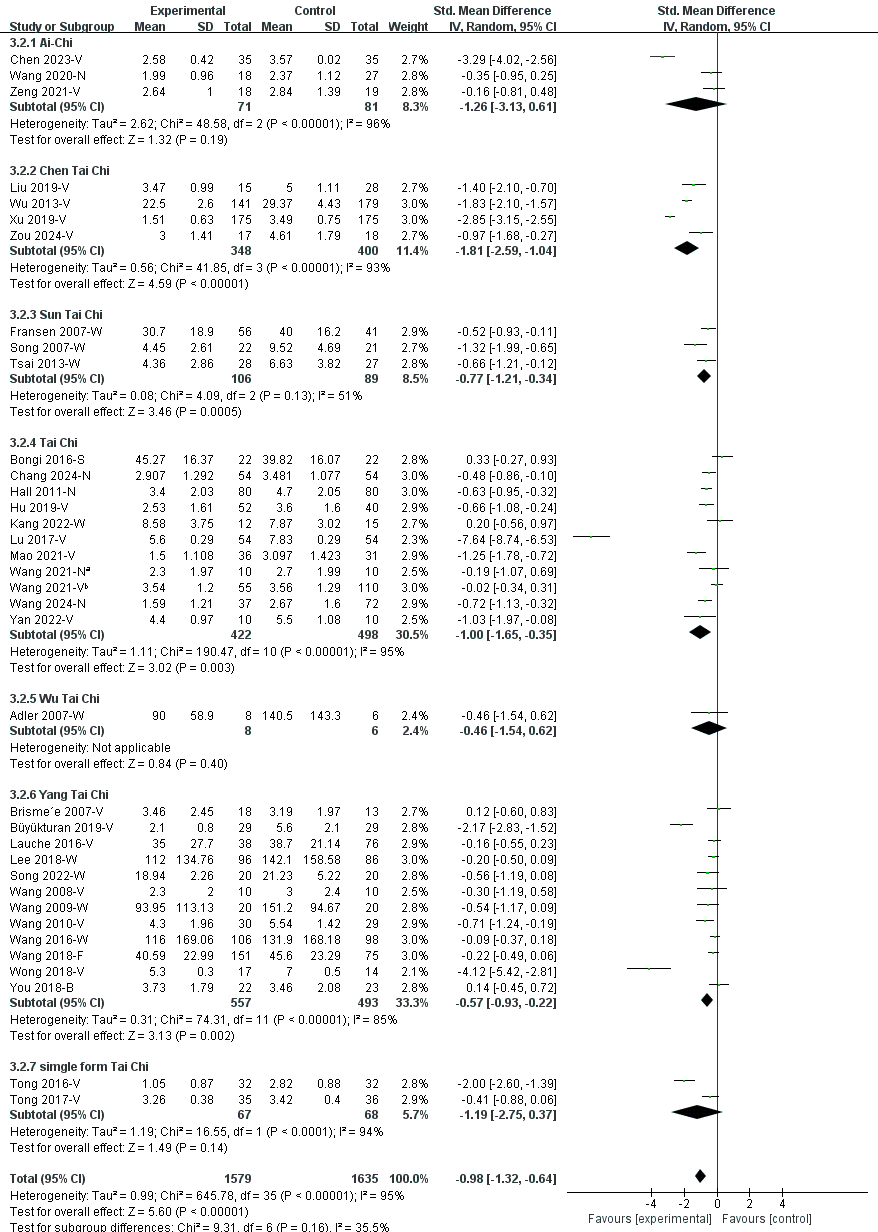


**Fig S2.** Exploratory subgroup analyses of Tai Chi on chronic musculoskeletal pain by Tai Chi types. Considerable heterogeneity was observed within subgroups; results are exploratory and should be interpreted with caution.

***Note***: B=Brief Pain Inventory, F=Fibromyalgia Impact Questionnaire, N=Numerical Rating Scale, S=Survey Short Form 36 Bodily Pain, V=Visual Analogue Scale, W=Western Ontario and McMaster Universities Osteoarthritis Index. Wang 2021-N^a^ (Ref. [33]) and Wang 2021-V^b^ (Ref. [25]) denote two separate studies.


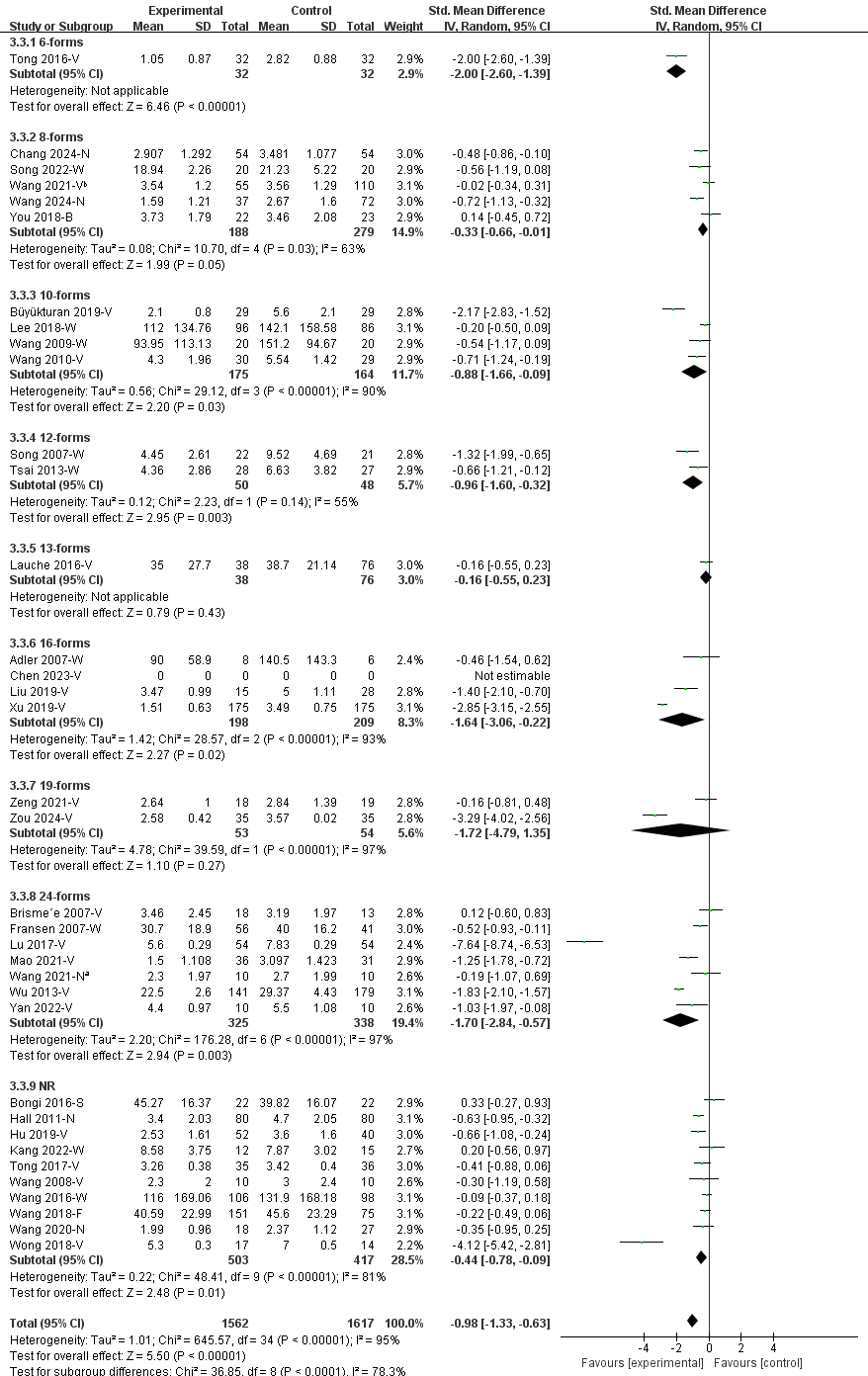


Fig S3. Exploratory subgroup analysis of Tai Chi on chronic musculoskeletal pain by Tai Chi forms. Results should be interpreted cautiously due to substantial heterogeneity and the limited number of studies in subgroups.

***Note***: B=Brief Pain Inventory, F=Fibromyalgia Impact Questionnaire, N=Numerical Rating Scale, NR= No Report, S=Survey Short Form 36 Bodily Pain, V=Visual Analogue Scale, W=Western Ontario and McMaster Universities Osteoarthritis Index. Wang 2021-N^a^ (Ref. [33]) and Wang 2021-V^b^ (Ref. [25]) denote two separate studies.
